# Oleic acid ameliorates palmitic acid induced hepatocellular lipotoxicity by inhibition of ER stress and pyroptosis

**DOI:** 10.1186/s12986-020-0434-8

**Published:** 2020-01-30

**Authors:** Xin Zeng, Min Zhu, Xiaohong Liu, Xuanmin Chen, Yujia Yuan, Lan Li, Jingping Liu, Yanrong Lu, Jingqiu Cheng, Younan Chen

**Affiliations:** Key Laboratory of Transplant Engineering and Immunology, NHFPC; Regenerative Medicine Research Center, West China Hospital, Sichuan University, No. 1, Keyuan 4th Road, Gao Peng Street, Chengdu, Sichuan 610041 People’s Republic of China

**Keywords:** Pyroptosis, ER stress, Lipotoxicity, Oleic acid, Palmitic acid

## Abstract

**Background:**

Pyroptosis is a novel programmed cell death. It is identified as caspase-1 dependent and characterized by plasma-membrane rupture and release of proinflammatory intracellular contents inculuding IL-1 beta and IL-18. Pyroptosis is distinct from other forms of cell death, especially apoptosis that is characterized by nuclear and cytoplasmic condensation and is elicited via activation of a caspase cascade. In pyroptosis, gasdermin D (GSDMD) acts as a major executor, while NLRP3 related inflammasome is closely linked to caspase-1 activation. Given that pyroptosis has played a critical role in the progression of non-alcoholic steatohepatitis (NASH), here, we investigated whether the regulation of pyroptosis activation is responsible for the protective role of monounsaturated oleic acids in the context of hepatocellular lipotoxicity.

**Methods:**

Human hepatoma cell line HepG2 cells were exposed to palmitic acid (PA) with or without oleic acids (OA) or/and endoplasmic reticulum (ER) stress inhibitor tauroursodeoxycholic acid (TUDCA) for 24 h. Besides, the cells were treated with the chemical ER stressor tunicamycin (TM) with or without OA for 24 h as well. The expressions of pyroptosis and ER stress related genes or proteins were determined by real-time PCR, Western blot or immunofluorescence. The morphology of pyroptosis was detected by acridine orange and ethidium bromide (AO/EB) staining. The release of IL-1 beta and tumor necrosis factor alpha (TNF-α) was determined by ELISA. Sprague–Dawley (SD) rats were fed with high fat diet (HFD) for 16 w, then, HFD was half replaced by olive oil to observe the protective effects of olive oil. The blood chemistry were analyzed, and the liver histology and the expressions of related genes and proteins were determined in the liver tissues.

**Results:**

We demonstrated that PA impaired the cell viability and disturbed the lipid metabolism of HepG2 cells *(P < 0.01)*, but OA robustly rescued cells from cell death *(P < 0.001)*. More importantly, we found that instead of cell apoptosis, PA induced significant pyroptosis, evidenced by remarkably increased mRNA and protein expressions of inflammasome marker NLRP3, Caspase-1 and IL-1beta, as well as cell membrane perforation driving protein GSDMD *(P < 0.05)*. Furthermore, we demonstrated that the PA stimulated ER stress was causally related to pyroptosis. The enhanced expressions of ER stress markers CHOP and BIP were found subcellular co-located to pyroptosis markers NLRP3 and ASC. Additionally,TM was able to induce pyroptosis like PA did, and ER stress inhibitor TUDCA was able to inhibit both PA and TM induced ER stress as well as pyroptosis. Furthermore, we demonstrated that OA substantially alleviated either PA or TM induced ER stress and pyroptosis in HepG2 cells *(P < 0.01)*. In vivo, only olive oil supplementation did not cause significant toxicity, while HFD for 32 w obviously induced liver steatosis and inflammation in SD rats *(P < 0.05)*. Half replacement of HFD with olive oil (a mixed diet) has remarkably ameliorated liver abnormalities, and particularly inhibited the protein expressions of either ER stress and pyroptosis markers *(P < 0.05)*.

**Conclusion:**

Palmitic acid induced predominant pyroptosis in HepG2 cells, and ER stress may be responsible for the induction of pyroptosis and subsequent cell death. Monounsaturated oleic acids were able to ameliorate hepatocellular lipotoxicity both in vitro and in vivo, and OA mediated inhibition of ER stress and pyroptosis may be the underlying mechanisms.

## Introduction

Non-alcoholic fatty liver disease (NAFLD) is characterized by steatosis at the first stage, and is able to progress to non-alcoholic steatohepatitis (NASH) or even ultimately cryptogenic cirrhosis and hepatocellular carcinoma [1, 2]. In the last decade, NAFLD has been considered as a serious public healthy burden worldwide [3]. Although lifestyle change would benefit improvement of hepatic steatosis, it is barely effective when NAFLD has progressed to NASH and further sever liver damage.

The pathogenesis of NAFLD is hypothesized to begin with abnormal accumulation of lipids in the liver due to a stress condition such as obesity and imbalanced nutrition uptake. Mechanistically, the associated signaling pathways include the accumulation of reactive oxygen species (ROS), endoplasmic reticulum (ER) stress, increased ceramide synthesis in hepatic cells, and so on [4]. It was commonly accepted that most of the above pathways lead to hepatocellular apoptosis (programmed cell death) and necrosis (non-programmed cell death) [5]. But more recently, it has been revealed that other types of programmed cell death, such as pyroptosis, may play an important role in NAFLD. Meanwhile, many studies agree with the important role of inflammation in the development and progression of NAFLD. Therefore, we hypothesize that pyroptosis is an better inflammatory link between NAFLD and NASH.

It is commonly accepted that, lipotoxicity is intimately associated with chronic inflammatory conditions, and immune cells infiltration in liver is a typical histologic character of NASH. Increasing evidence indicates that NLRP3 inflammasome is particularly active in livers of mice and humans with NASH. Furthermore, it is found that upon ER stress, activated IRE1α activates protein IkB and JNKs, which implicates the transcriptional activation of pro-inflammatory and pro-apoptotic pathways [6]. Thus, we hypothesized that inflammation, particular NLRP3 inflammasome may be the critical link between ER stress and eventually cell damage in the context of hepatic lipotoxicity. Given that, we focused on pyroptosis, a lately distinguished inflammatory programmed cell death.

More recently, it has become clear that other types of programmed cell death, such as necroptosis and pyroptosis, perhaps play unexpected roles in NASH. Apoptosis, a caspase-dependent pathway, is characterized by nuclear condensation and cellular fragmentation into apoptotic bodies that are phagocytosed and degraded by macrophages [7]. This non-lytic pathway has minimal effects on the surrounding cells. In contrast, lytic cell death is highly inflammatory, and includes not only necrosis but also the programmed cell death pathways necroptosis and pyroptosis. Pyroptosis, an osmotic lytic cell death, is most recently described as a downstream of inflammasome activation. Though pyroptosis is morphologically similar to necrosis, leading to membrane rupture and/or pore formation, it is exactly a programmed cell death similar to apoptosis, while the differences exist in its distinct features of caspase-1 dependence and initiation of strong inflammatory response by cleavaging proinflammatory cytokines pro-IL-1 beta and pro-IL-18.

The molecular mechanisms that trigger pyroptosis activation in NAFLD are still elusive. Increasing evidence has identified crosstalk between steatosis and cell death signaling, in which ER stress is distinguished as a crucial link between lipotoxicity and cellular damage. The imbalance between the ER protein load and ER folding capacity causes ER stress, leading to the hyperactivation of the unfolded protein response (UPR), which results in cell dysfunction and death. However, whether ER stress is associated to pytoptosis in NAFLD/NASH progression is unknown.

Dietary fatty acids are important in NAFLD development, while the different species of dietary fatty acids contribute differently to the liver lipotoxicity in the pathogenesis of NAFLD. The most abundant saturated fatty acid present in the diet and serum is palmitic acid (PA, 16:0) [8], that is able to trigger lipotoxicity in β cells [9], hepatocytes [10], muscular cells [11], and so on. Contrarily, unsaturated fatty acids, including monounsaturated fatty acids (MUFA), are much nontoxic, and are proven able to combat PA induced lipotoxicity [12–14]. Human studies indicate that MUFA-rich diet has protective effects in cardiovascular risk, NAFLD and diabetes [15–17]. Oleic acid (OA, 18:1 *n* = 9) is a typical MUFA, and is the predominant component in olive oil (70–80%). The beneficial effects of olive oil as food supplement are widely investigated [18–20], but the molecular mechanisms remain poor understood.

In the present study, we are aiming to explore the roles of pyroptosis and ER stress in the context of saturated fatty acids induced lipotoxicity, and we asked if the protective effects of monounsaturated oleic acid or olive oil on hepatocellular dysfunction in NASH is causally related with its amelioration of ER stress and pyroptosis.

## Materials and methods

### Animals and diets

Male Sprague–Dawley (SD) rats (100–150 g body weight) were purchased from DASHUO animal company (SCXK (CHUAN) 2015–30, Chengdu, China). Rats were housed in the animal care facilities at an appropriate temperature (21–25 °C) and humidity (40–70%) with 12 h light/dark cycle. After a two-week adaptive period, rats were first fed with either chow diet (containing 4% fat, control group) or high fat diet (HFD, containing 10% fat and 2% cholesterol) for 16 w to induce hepatic steatosis. Thereafter, the control group was randomly divided to two groups. One group was continuously fed with chow diet (Control group), another one was switched to supplementation with extra virgin olive oil (Baena, Spain, OA group). Additionally, the HFD fed animals were randomly divided to two groups as well. One group was continuously fed with HFD for a further 16 w to develop NASH (HFD group), and for another group, half of the high fat food was replaced by olive oil, so the animals were fed with the mixed diet for a further 16 w (HFD/OA group). Importantly, the food calorie in all the groups, except for Control group, was equal as approximately 83 kcal/rat/day. Body weight and random plasma glucose were monitored every week, and the food intake was recorded accordingly. At the end of the treatments, rats were anesthetized with 10% chloralhydrate and the liver samples were carefully collected for later measurements. Each group has five different individuals (*N* = 5). All of the experimental procedures were approved by the Institutional Animal Care and Use Committee (IACUC) of Sichuan University.

### Blood biochemical analysis

Serum alanine transaminase (ALT), aspartate aminotransferase (AST), triglyceride (TG), total cholesterol (TC), high-density lipoprotein cholesterol (HDL-C) and low-density lipoprotein cholesterol (LDL-C) were detected by an auto-analyzer (Cobas 6000 c501, Roche Diagnostics, Switzerland).

### Liver histology

Liver samples were sliced into 5 μm sections and were routinely stained for hematoxylin and eosin (H&E). The gradations of lobular involvement by steatosis, inflammation and ballooning degeneration, and portal inflammation are the components from which the global grade of NAFLD activity is derived [21].The grade of lobular inflammation was assessed on the basis of the NAFLD activity score and graded from 0 to 3 based on inflammatory foci per 20 × magnification.

### Preparation of stock solutions of fatty acids

Dissolve 0.0641 g PA (Aladdin, China) and 0.0706 g OA (Aladdin, China) in 2.5 ml 100% ethanol, respectively, to reach a final concentration of 100 mM. These solutions were then mixed with 22.5 ml 20% fatty acid-free BSA (Solarbio, China) in phosphate-buffered saline (PBS) at 50 °C for 1 h, yielding a final stock solution of 10 mM. A control 18% BSA solution was prepared by mixing 2.22 ml 100% ethanol with 20 ml 20% fatty acid-free BSA. All stock solutions were stored in − 20 °C.

### Cell culture

Human hepatoma cell line HepG2 cells (purchased from ATCC) were cultured in high glucose DMEM supplemented with 10% fetal bovine serum (FBS) and 1% antibiotics (100 U/ml penicillin and 100 μg/ml streptomycin) at 37 °C, respectively. When reaching about 80–90% confluence, cells were digested and cultured overnight in 96-well plates or 6-well plates.Then, the medium was changed to fresh medium containing 10% FBS, and different treatments.Tauroursodeoxycholic acid (TUDCA) and 4-phenylbutyrate (4-PBA) are chemical ER inhibitors, purchased from MedChemExpress (China). Tunicamycin (TM) is chemical ER stressor, purchased from Santa Cruz Biotechnology (Santa Cruz, CA, USA). After treatments, the medium was collected and the cells were harvested for subsequent assays.

### Cell viability

Cells were cultured in 96-well plate overnight at a density of 5000 cells/well for HepG2. After the treatments, cells were washed three times with PBS and cell viability was measured using a Cell Counting Kit-8 (CCK8,DOJINDO, Japan), according to the manufacturer’s instruction.

### Cell apoptosis

Cells were cultured overnight in 6-well plate with 1 × 10^5^ cells/well for HepG2. After 24 h exposure to PA and/or OA, cells were harvested and subjected to annexin V-PI (BD, USA) binding, then detected by flow cytometry (FCM).

### Lipid accumulation

Cells were cultured for 24 h in a 96-well plate and then exposed to the indicated treatments. Cellular lipid accumulation was visualized by Oil Red O staining after 24 h treatment.

### Enzyme-linked immunosorbent assay (ELISA)

Cell culture supernatants were measured for IL-1 beta (ab46052; abcom), and TNF-α (A098526-48 T; affandi) using ELISA kits according to the manufacturer’s instructions.

### RNA isolation and quantitative real-time PCR

Total RNA was isolated from cultured cells or fresh liver samples using Trizol reagent (Ambion, USA), and 1.0 μg RNA was reverse-transcribed into cDNA using a high capacity cDNA Synthesis Kit (Vazyme, China) according to the manufacturer’s protocol. Realtime PCR was performed to assess gene expression in a Bio-Rad QPCR Machine using SYBR Green master mix (Vazyme, China). Each sample was amplified in triplicate, and the expression of β-actin was used as an internal control for every PCR assay.

### Western blot analysis

Total protein was extracted from cultured cells or fresh liver samples using RIPA lysis buffer. BCA Protein Assay Kit (Cwbio, China) was used to measure protein concentrations. Protein extracts (60 μg) were separated on a 10% SDS–PAGE gel and transferred to a 0.2 μm PVDF membrane. Antibodies against cleaved Caspase3 (#9668, CST, MA, USA) and Caspase9 (#9746 CST, USA), C/EBP homologous protein (CHOP)/(Santa, USA), Glucose 2 regulated protein 78 kD (GRP78) /(abs130538a, Absin, China), NOD-like receptor family pyrin domain containing 3 (NLRP3)/(DF7438, Affinity, China), gasdermin D (GSDMD) and gasdermin-N domain (GSDMD-N)/(20,770–1-AP, proteintech, China), Pro-Caspase-1 (WL02996,wanlei,China) and cleaved Caspase-1 (p20)/(WL02996a, wanlei, China), and mature IL-1 beta (AF4006, Affinity, China) were used for immunoblotting. β-actin (Abcam, USA) was used as a loading control. The immunoblots were visualized using a ChemiDoc™ imaging system (Bio-Rad, USA).

### Immunofluorescence analyses

Immunofluorescence staining was performed to detect the in situ expression of target proteins via specific antibodies. DAPI was used to stain the nucleus of cells. Photographs were blindly taken at random fields under a fluorescence microscope (ZEISS Axio vert.A1, Germany). Representative images are shown in the figures.

### Acridine orange and ethidium bromide (AO/EB) staining

The cells were treated with BSA, PA or PA plus OA for a further 24 h, then subjected to AO/EB staining (100 μg/ml of AO and 100 μg/ml of EB mixed in PBS, Aladdin, China). The fluorescence of cells was observed under fluorescent microscope.

### Statistical analysis

Experiments were performed at least three times and quantitative data are expressed as mean ± SD. GraphPad Prism 6 was used for statistical analyses. Data were evaluated with a 2-tailed, unpaired Student’s t test or compared by one-way analysis of variance. A value of *P* < 0.05 was considered statistically significant.

## Results

### Oleic acid protected HepG2 cells from palmitic acid induced lipotoxicity

**Fig. 1:**
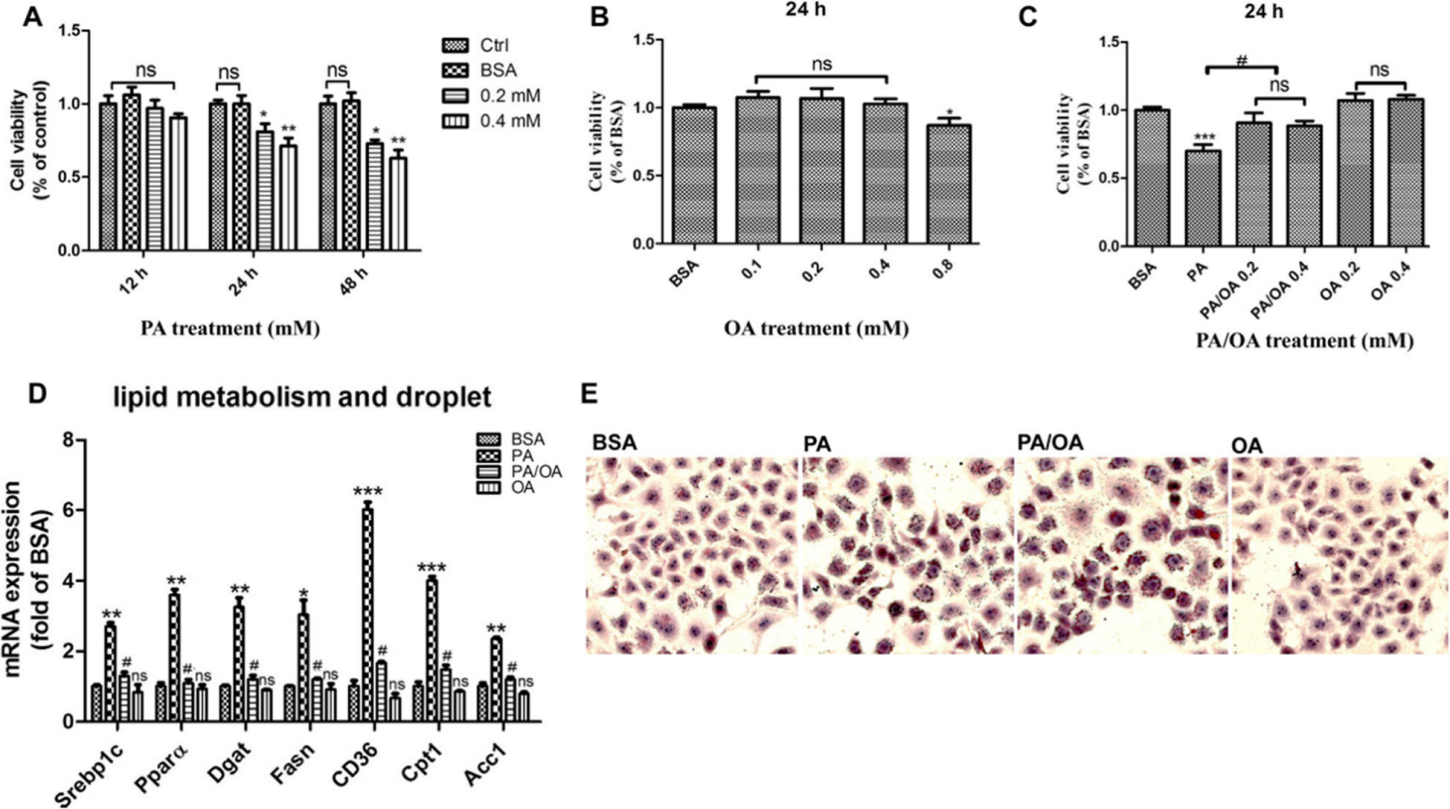
Oleic acid protected HepG2 cells from palmitic acid induced Lipotoxicity. Viability of HepG2 cells was assessed using the CCK8 assay. **a**. and **b**. Alternatively, cells were treated with PA or OA alone for 12 h,24 h or 48 h. **c**. Cells were concomitantly incubated with PA and OA for 24 h. **d**. HepG2 were treated with 0.4 mM PA, 0.2 mM OA or combination of 0.4 mM PA plus 0.2 mM OA (PA/OA). The mRNA expression of key genes governing lipid metabolism were detected after 24 h treatment, and β-ACTIN was used as an internal control; **e**. Cells were stained with Oil Red O and lipid accumulation was visualized under a microscope at 200 × magnification after 24 h treatment. The data are presented as means ± SD for 3–5 biological replicates; *P < 0.05, **P < 0.01, *** *P* < 0.001vs. BSA;#*P* < 0.05,vs. PA; ns no significant differences between two connected groups

To determine the cytotoxicity of PA on hepatocytes. HepG2 cells were exposed to different concentrations (0.2 and 0.4 mM) of PA for 12 h, 24 h or 48 h. Cell viability was measured by CCK8 assay. Consistent to the previous studies, PA markedly deteriorated cell viability in a dose and time dependent manner *(P < 0.05, P < 0.01)* (Fig. 1a). In contrast to PA, monounsaturated OA showed no toxicity at a concentration up to 0.4 mM after 24 h exposure (Fig. 1b). However, OA was able to neutralize PA induced Lipotoxicity in a dose dependent manner. Surprisingly, a 24 h concomitant incubation of PA and OA at a mole ratio of 2:1 completely restored the HepG2 viability *(P < 0.05, P < 0.001)* (Fig. 1c). The disturbance of cellular lipid metabolism by PA is supposed to be responsible to its lipotoxicity. The results of genes expressions indicated that PA exposure increased the mRNA expression of a couple of genes governing lipid metabolism including *Srebp1c, Pparα, Dgat, Fasn, Cd36, Cpt1* and *Acc1,* while these up-regulations have not been found in OA treated cells, but were significantly diminished by OA co-supplementation with PA *(P < 0.05, P < 0.01, P < 0.001)* (Fig. 1d). Nevertheless, PA plus OA group exhibited more lipid accumulation in HepG2 cells than either control or exclusive PA group, evidenced by oil red O staining (Fig. 1e), which suggested that the production of neutral lipids may not be directly responsible for cellular lipotoxicity. Summarily, these results demonstrated that OA was able to powerfully combat PA induced lipotoxicity.

### Apoptosis is not the main form of cell death caused by palmitic acid induced lipotoxicity

**Fig. 2:**
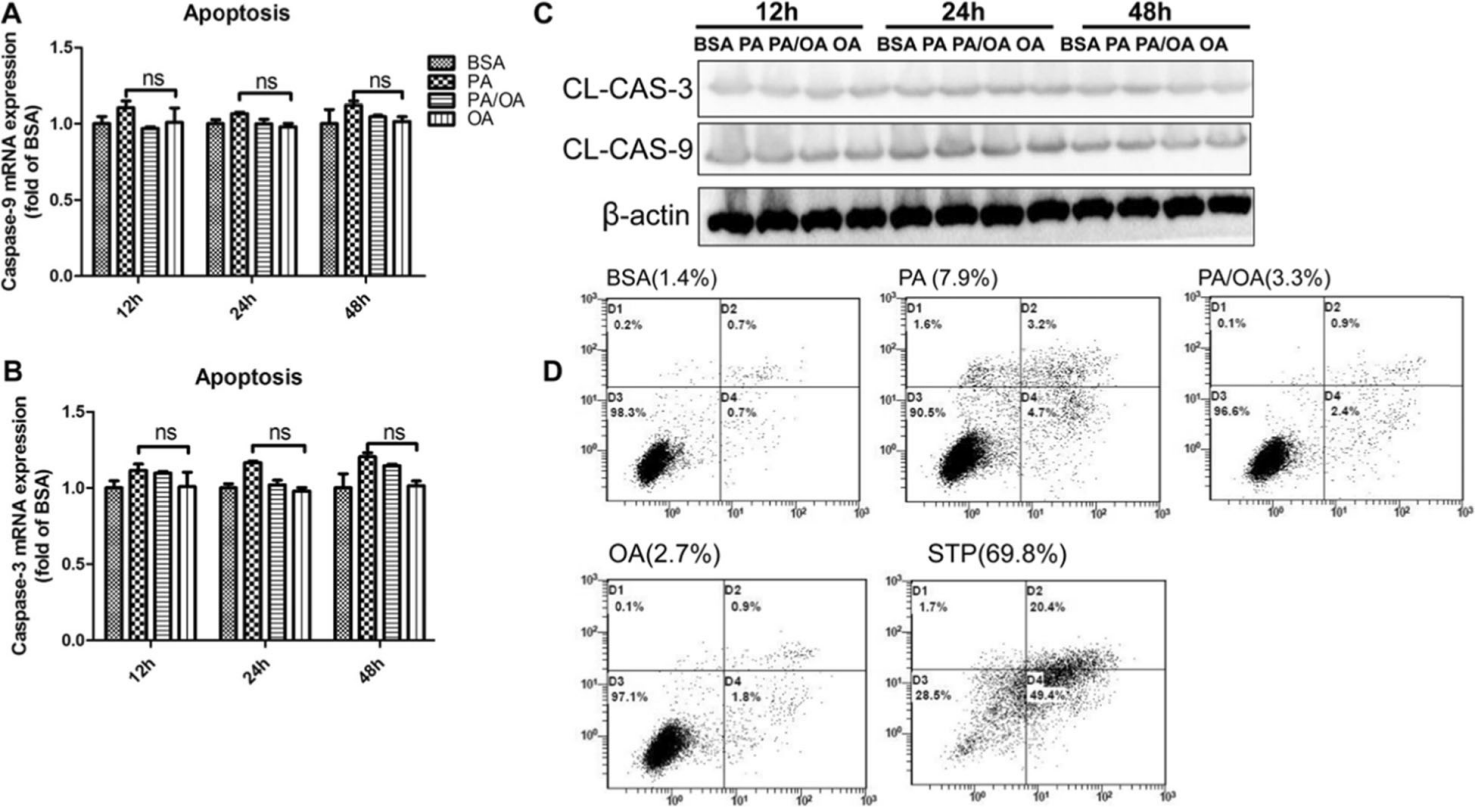
Apoptosis is not the main form of cell death caused by palmitic acid induced Lipotoxicity. HepG2 were treated with 0.4 mM PA, 0.2 mM OA or combination 0.4 mM PA plus 0.2 mM OA (PA/OA). **a** and **b.** The mRNA expression of key genes governing apoptosis were detected after 24 h treatment, and β-ACTIN was used as an internal control. **c**. Representative western blots of Cleaved-caspase3/9 after 24 h treatment, and β-ACTIN was used as a protein-loading control. **d**. Apoptosis assay using FCM with AV/PI staining after 24 h treatment. The numbers at the lower or upper right indicate the percentage of sum of early and late apoptotic cells. The data are presented as means ± SD for 3–5 biological replicates; *P < 0.05, **P < 0.01, ***, *P* < 0.001vs. BSA; ns no significant differences between two connected groups

Lipotoxicity will ultimately results in cell death, and consequent organ dysfunction. To elucidate this process, we firstly determined if PA induced lipotoxicity was associated to cell apoptosis. HepG2 cells were exposed to 0.4 mM PA or plus 0.2 mM OA for 12 h, 24 h or 48 h. Unexpectedly, neither PA exposure or OA intervention changed the mRNA expression of a couple of apoptotic genes including *caspase-3* and *caspase-9* (Fig. 2a, b). Meanwhile, activated-caspase-3/− 9, the most important terminal cleavage enzymes in apoptosis were barely altered in the results of Western blot *(P < 0.05)* (Fig. 2c). Furthermore, cell apoptosis was analyzed via flow cytometry with annexin V-PI staining. Quantification of apoptotic cells showed that PA has not provoked dramatic cell apoptosis (7.9%) after 24 h exposure, which was inconsistent to the marked decrease of cell viability seen in CCK8 detection. However, the typical apoptosis inducer staurosporine induced dramatic cell apoptosis (69.8%) as a positive control (Fig. 2d). On the other hand, OA addition still showed minimal protection, decreasing the apoptotic cells from 7.9 to 3.3% in this context. Taken together, these results suggested that apoptosis may be not the main form of cell death caused by palmitic acid induced lipotoxicity.

### Palmitic acid induced pyroptosis activation,and oleic acid protected HepG2 cell against pyroptosis

**Fig. 3:**
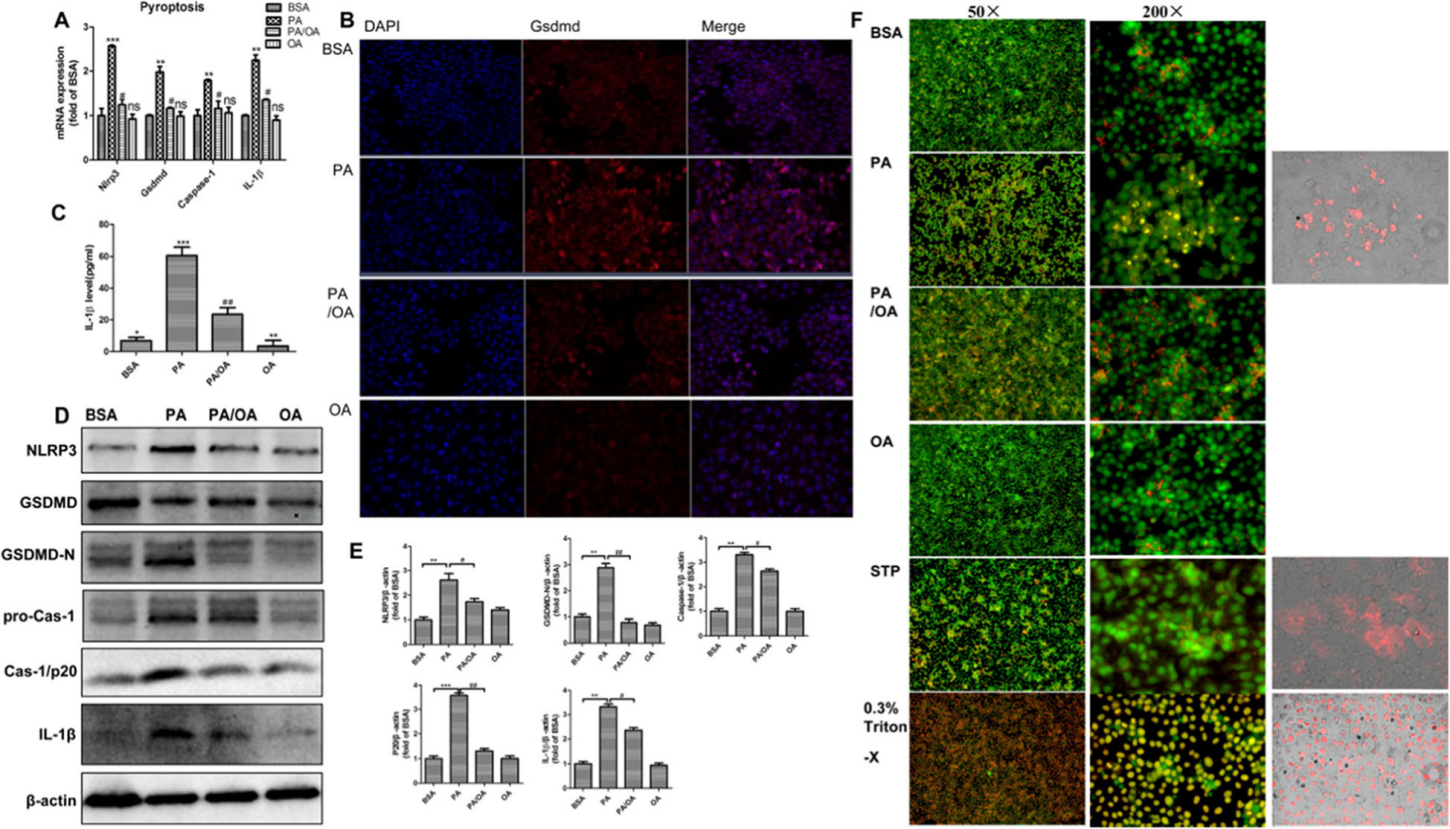
Palmitic acid induced pyroptosis activation,and oleic acid protected HepG2 cell against pyroptosis. HepG2 were treated with 0.4 mM PA, 0.2 mM OA or their combination (PA/OA). **a.** The mRNA expressions of key genes governing pyroptosis were detected after 24 h treatment, and β-ACTIN was used as an internal control. **b.** HepG2 cells were stained with anti-GSDMD (red) antibody and DAPI, and then visualized under a microscope at 200× magnification after 24 h treatment. **c**. Cell supernatants were analyzed for IL-1β secretion by ELISA. **d** and **e**. Representative western blots of NLRP3, GSDMD/−N, pro-CAS-1, P20 and IL-1β after 24 h treatment, and β-ACTIN was used as a protein-loading control. **f**. The morphology of pyroptotic cells was visualized by AO/EB staining. All groups were visualized under a microscope at 50× and 200× magnification after 24 h treatment. The data are presented as means ± SD for 3–5 biological replicates; **P < 0.01, ***, *P* < 0.001vs. BSA;#*P* < 0.05,##*P* < 0.01,vs. PA; ns no significant differences between two connected groups

To identify the foremost pathway in PA-induced cell death, we examined representative molecular markers of pyroptosis. The qPCR results showed that 0.4 mM PA significantly upregulated the mRNA expression of *Nlrp3*, the typical marker of inflammasome after 24 h treatment, which paralleled the upregulations of *Gsdmd, Caspase-1* and *Il-1 beta* upon pyroptosis. Interestingly, only OA treatment showed no impact on these gene, but PA combined with OA substantially attenuated the effects of PA on inflammasome and pyroptosis in HepG2 cells*(P < 0.05,P < 0.01,P < 0.001)* (Fig. 3a). Consistently, the Western blot results indicated that PA treatments substantially increased the protein levels of pyroptosis markers. The most distinct up-regulations presented on NLRP3, GSDMD/−N (the activated form of GSDMD), Caspase-1, p20 (the activated form of Caspase-1), and IL-1 beta. As expected, these alterations were substantially abolished by OA supplement(*P < 0.05,P < 0.01,P < 0.001*) (Fig. 3d, e). Essentially, gasdermin D(GSDMD) is critical to drive pyroptosis and control IL-1 beta release. To convince the activation of pyroptosis pathway, we detected the in situ expression of GSDMD as well as the release of mature IL-1 beta. Consistent to the mRNA upregulation, immunofluorescence and ELISA confirmed that PA profoundly provoked the expression of GSDMD and release of IL-1 beta in HepG2 cells. However, OA was able to ablate this process (Fig. 3b, c).

It is demonstrated that pyroptosis features pore formation in the plasma membrane, cells welling and rupture of the membrane, causing massive leakage of cytosolic contents. To visualize this morphological alteration of cells undergoing pyroptosis and distinguish it from apoptosis, we used AO/EB staining to specifically observe the changes of cell permeability under microscope. The results suggested that PA treated cells were obviously swelling, compared with cells of other groups. More importantly, both PA driven pyroptotic cells and 0.3%Triton X 100-treated cells exhibited massive red fluorescence entering into cells by EB staining, but staurosporine-induced apoptotic cells were characterized by red fluorescence surrounding the cell membrane as well as no distinct cell swelling (Fig. 3f). Additionally, the combination of PA and OA significantly inhibited the process of pyroptosis, which convinced the protective role of OA in HepG2 cells (Fig. 3f).

Overall, these results indicated that PA might drive NLRP3 inflammasome-mediated pyroptosis activation, and OA was able to alleviate PA-induced pyroptosis in HepG2 cells.

### Palmitic acid induced ER stress was associated with pyroptosis activation,and oleic acid protected HepG2 cells from ER stress

**Fig. 4:**
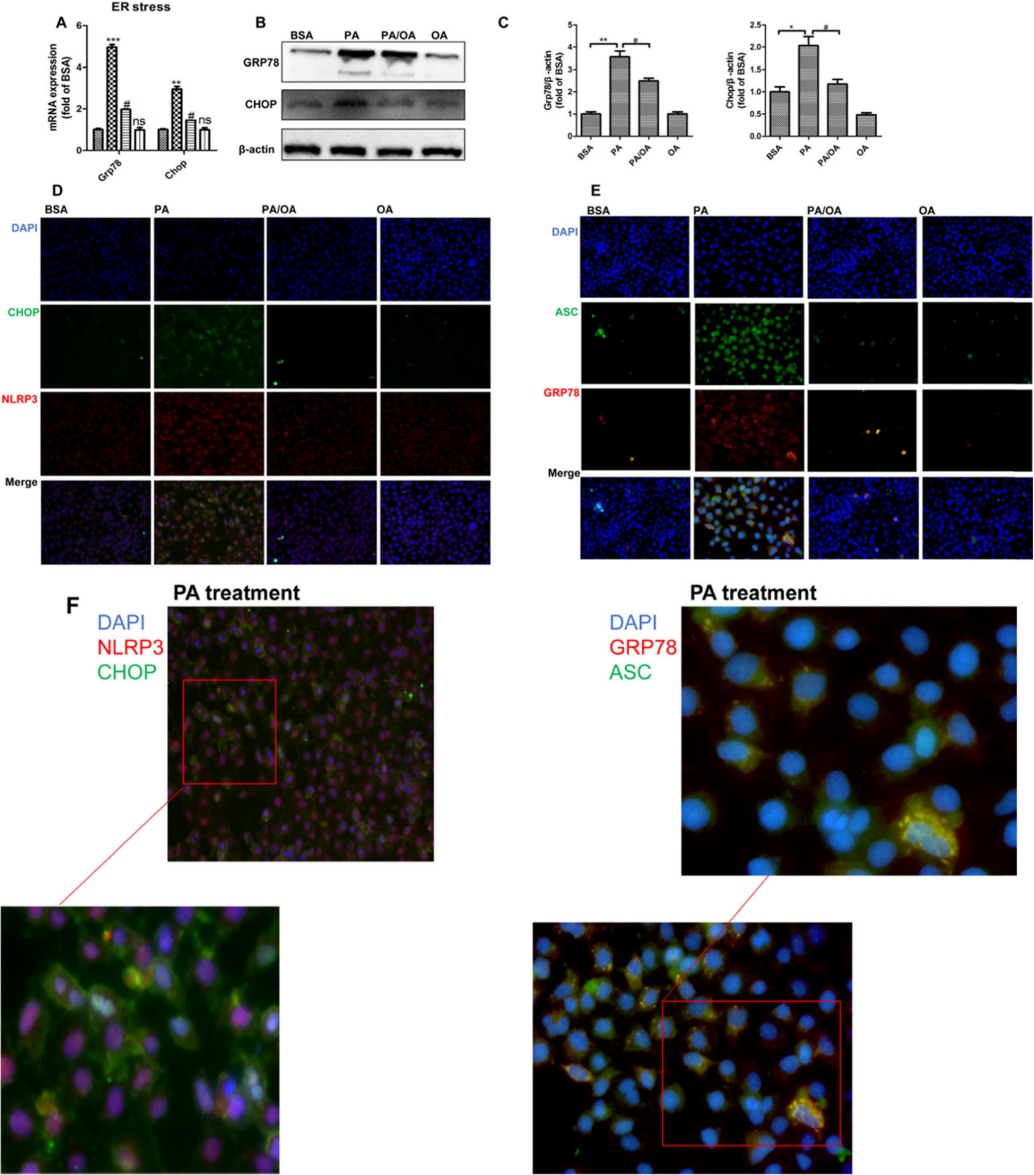
Palmitic acid induced ER stress was associated with pyroptosis activation, and oleic acid protected HepG2 cells from ER stress. HepG2 were treated with 0.4 mM PA, 0.2 mM OA or their combination (PA/OA). **a**-**b**. The mRNA expressions (**a**) and protein expressions (**b**) of key genes governing ER stress were detected after 24 h treatment, and β-ACTIN was used as an internal control for qPCR and Western Blot, respectively. **c**. Representative western blots of GRP78 and CHOP after 24 h treatment, and β-ACTIN was used as a protein-loading control. **d**. HepG2 cells were exposed to PA or OA and subsequently labeled by immunofluorescence with anti-NLRP3 (red) and anti-CHOP (green) antibodies, respectively. **e**. All groups labeled for GRP78 (red) and ASC (green) as well. **f**. HepG2 cells were exposed to PA and was visualized under a microscope at 400× magnification after 24 h treatment. Inset shows zoomed of indicated region. The data are presented as means±SD for 3–5 biological replicates; *P < 0.05, **P < 0.01, vs. BSA; ^#^*P* < 0.05, vs. PA; ns no significant differences between two connected groups

A good amount of studies have demonstrated that ER stress has played a key role in lipotoxicity related cell death, and our previous studies found that OA was able to alleviate lipotoxicity induced ER stress in either hepatocyte or islets. To explore whether ER stress is linked to the PA mediated pyroptosis, as well as the protection of OA, we detected the ER stress markers in HepG2 cells. Consistent to previous results, PA obviously upregulated both mRNA and protein levels of ER stress markers GRP78 and CHOP, and OA remarkably inhibited these alterations*(P < 0.05,P < 0.01,P < 0.001)* (Fig. 4a-c).

Biochemical evidence suggests that NLRP3 is mainly localized on ER membrane. Upon inflammasome activation, ASC(apoptosis-associated speck-like protein containing card) oligome-rizes to form the so-called ASC speck [22]. To assess the relationship between ER stress and the NLRP3 inflammasome under the challenge, we analyzed the co-localization of CHOP and NLRP3, as well as GRP78 and ASC in HepG2 cells. After exposure to PA for 24 h, NLRP3 was substantially elicited, which was accompanied with the strong expression of CHOP, while OA restrained the connection. Additionally, stimulation of the NLRP3 via PA was sufficient to assemble the inflammasome, resulting in ASC speck formation, which was found co-localized with the increased expression of GRP78 in the cytoplasm of HepG2 cells (Fig. 4e). Importantly, OA treatment was able to robustly inhibit the increase of both the ER stress markers CHOP/GRP78 and the inflammasome marker NLRP3, and ASC speck formation was also significantly abolished by OA (Fig. 4f). Taken together, our results provided evidence that PA stimulated pyroptosis was closely related to ER stress, and OA was capable to inhibit the progression of ER stress as well pyroptosis, which may be responsible for its powerful protective role in lipotoxicity of HepG2 cells.

### ER stress induced NLRP3 inflammasome -mediated pyroptosis activation

**Fig. 5:**
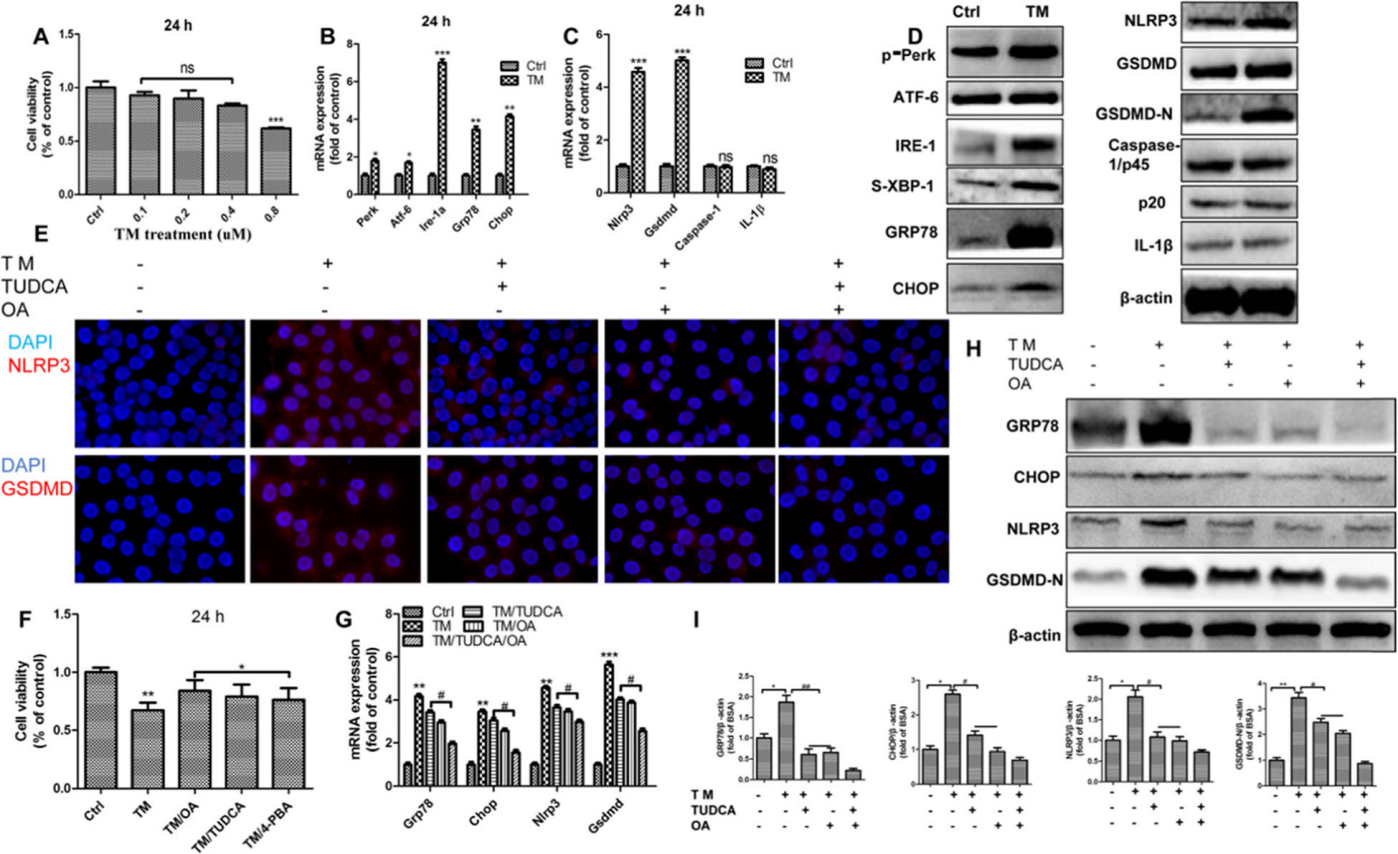
ER Stress induced NLRP3 inflammasome -mediated pyroptosis activation. The ER stress in HepG2 cells were elicited by chemical ER stressor tunicamycin (TM) for 24 h. **a**. Cell viability of cells was assessed using the CCK8 assay. **b**. and **c**. The mRNA expression of key genes governing ER stress and pyroptosis were detected after 24 h treatment of 0.8 uM TM, and β-ACTIN was used as an internal control. **d**. Representative western blots of ER stress and pyroptosis makers, and β-ACTIN was used as a protein-loading control. **e**. HepG2 cells were exposed to TM and plus with TUDCA or OA followed by labeling with anti-GSDMD or anti-NLRP3 antibody and were visualized under a microscope at 400× magnification after 24 h treatment. **f**. Cells were exposed to TM, or plus with TUDCA, 4-PBA or OA, and cell viability was assessed using the CCK8 assay. **g**. The mRNA expression of key genes governing ER stress and pyroptosis were detected after 24 h treatment, and β-ACTIN was used as an internal control. **h**. and **i**. Representative western blots of GRP78, CHOP, NLRP3 and GSDMD-N after 24 h treatment, and β-ACTIN was used as a protein-loading control. The data are presented as means ± SD for 3–5 biological replicates; *P < 0.05, **P < 0.01, ***P < 0.001vs. BSA;#*P* < 0.05, ##*P* < 0.01,vs. TM; ns no significant differences between two connected groups

ER stress can be chemically induced by some ER stressors, such as TM, a N-acetylglucosamine inhibiting protein glycosylation. In order to elucidate the relationship between ER stress and pyroptosis, HepG2 cells were exposed to different concentrations of TM for 24 h. We found that 0.8 uM TM declined the cell viability for approximately 30%, which was close to the cellular toxicity of 0.4 mM PA *(P < 0.001)*(Fig. 5a). As expected, the mRNA levels of UPR genes as well as the protein levels of ER stress markers were significantly increased after 24 h stimulation of TM *(P < 0.05,P < 0.01, P < 0.001)*(Fig. 5b,d). The most distinct upregulations presented on p-PERK, ATF6, IRE-1, sXbp-1, GRP78 and CHOP (Fig. 5d). Importantly, ER stressor significantly increased both gene and protein expressions of pyroptotic markers NLRP3 and GSDMD (Fig. 5c, d), which was convinced by the greatly elevated immunofluorescence of anti-NLRP3 and anti-GSDMD antibodies in HepG2 cells as well (Fig. 5e). In conclusion, these results revealed that ER stressors-induced ER stress might lead to NLRP3 inflammasome -mediated pyroptosis activation.

### Oleic acid abrogated pyroptosis in HepG2 cells through inhibiting ER stress

To understand the mechanism that underlies the regulation of ER stress and the NLRP3 inflammasome by OA, we hypothesized that OA is capable to alleviate ER stress, which leads to the inhibition of pyroptosis. We found that the combination of OA and TM robustly restored the cell viability to normal level in HepG2 cells *(P < 0.05, P < 0.01)* (Fig. 5f). Furthermore, the results from qPCR and Western blot demonstrated that OA markedly reduced the TM induced upregulations of ER stress markers GRP78/CHOP as well as pyroptosis markers NLRP3/GSDMD (*P < 0.05, P < 0.01, P < 0.001*) (Fig. 5g-i). It suggested that OA is an effective ER stress inhibitor, which contributes to its suppressive effects on pyroptosis. To give more evidence to this hypothesis, we used a chemical chaperones against ER stress, tauroursodeoxycholic acid (TUDCA), to investigate if the chemical ER stress inhibitors can blunt the activation of pyroptosis as OA did. The results showed that the effects of TUDCA on TM induced ER stress and pyroptosis were quite similar with OA, and combination of TUDCA and OA showed even synergetic protective effects (Fig. 5f-i). Therefore, our results demonstrated that the ER stress facilitated the activation of NLRP3 inflammasome, and OA was able to combat pyroptosis by suppressing ER stress.

### ER stress inhibitor abrogated palmitic acid induced pyroptosis activation

**Fig. 6:**
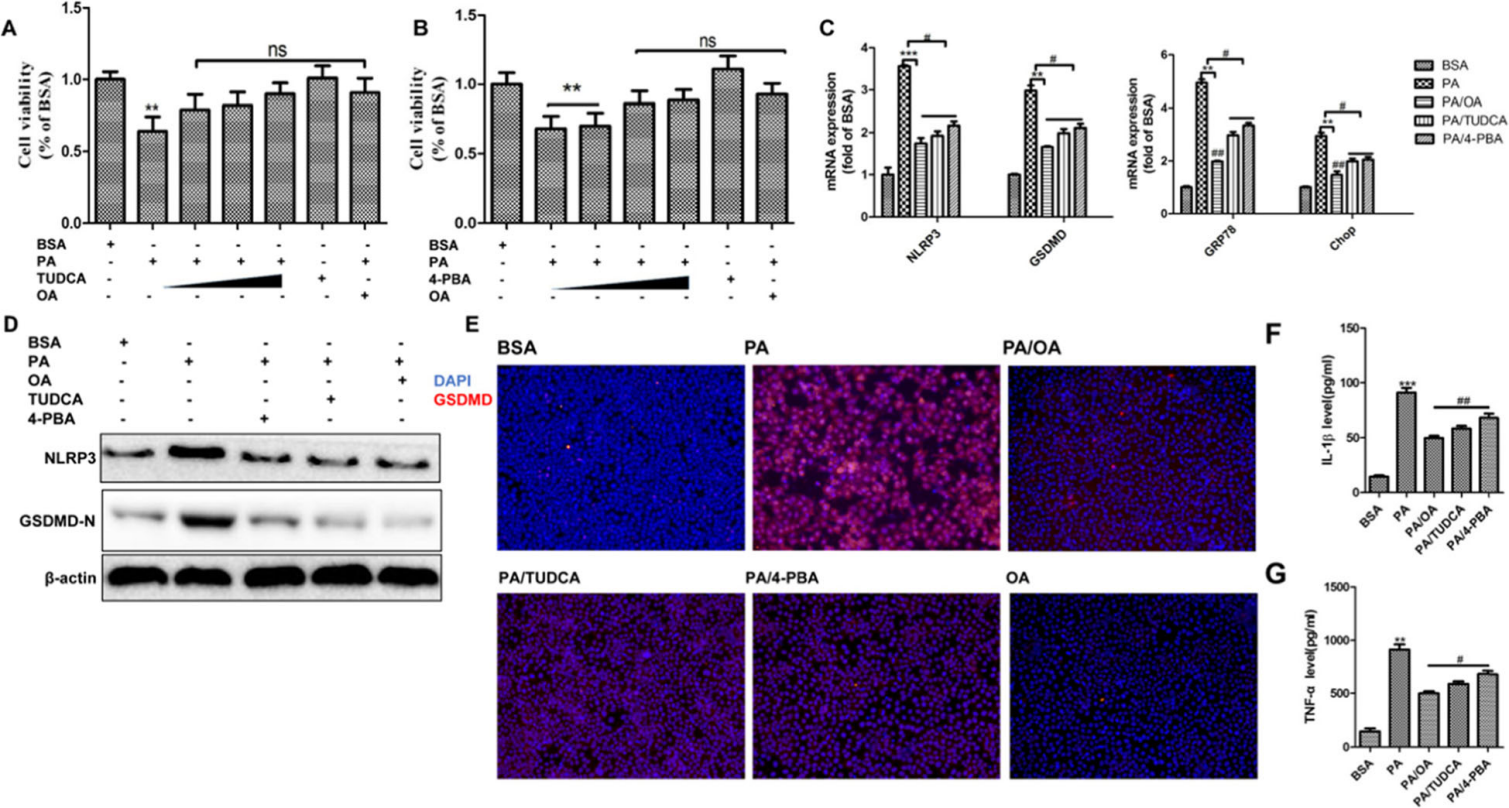
Oleic acid abrogated pyroptosis in HepG2 cells through inhibiting ER stress. The ER stress in cells were inhibited by chemical chaperones 4-phenylbutyric acid (4-PBA) or tauroursodeoxycholic acid (TUDCA) for 24 h against ER stress. **a**. and **b**. Cell viability of HepG2 cells was assessed using CCK8 assay. **c**. The mRNA expression of key genes governing pyroptosis were detected after 24 h treatment, and β-ACTIN was used as an internal control. **d**. Representative western blots of NLRP3 and GSDMD-N after 24 h treatment, and β-ACTIN was used as a protein-loading control. **e**. HepG2 cells were exposed to PA or plus OA, TUDCA or 4-PBA followed by labeling with anti-GSDMD (red) antibody and DAPI staining, and were visualized under a microscope at 100× magnification after 24 h treatment. **f**. and **g**. Cell supernatants were analyzed for IL-1β and TNF-α secretion by HepG2 cells by ELISA. The data are presented as means ± SD for 3–5 biological replicates; *P < 0.05, **P < 0.01, and ***P < 0.001vs. BSA;#*P* < 0.05, ##*P* < 0.01vs.PA;ns no significant differences between two connected groups

To further convince the link between ER stress and pyroptosis in the context of PA-induced lipotoxicity, we tested if chemical ER inhibitors were able to abrogate the activation of PA-mediated pyroptosis. HepG2 cells were exposed to 0.4 mM of PA or plus 0.2 uM TUDCA or 4-PBA for 24 h. We found that ER stress inhibitors showed no toxicity after 24 h exposure *(P < 0.01)* (Fig. 6a, b). However, they were able to neutralize PA induced lipotoxicity and completely restore the cell viability in a dose dependent manner (Fig. 6a, b). Consistently, TUDCA or 4-PBA treatment attenuated the effects of PA on mRNA and protein levels of NLRP3, GSDMD, GRP78 and CHOP (*P < 0.05, P < 0.01, P < 0.001*) (Fig. 6c, d). After TUDCA or 4-PBA treatments, the in situ expressions of GSDMD was remarkably decreased in HepG2 cells as well, which was similar to that of OA treatment (Fig. 6e). In addition, when PA induced-ER stress was compromised by OA or ER stress inhibitor, the releases of IL-1 beta as well as tumor necrosis factor α(TNF-α)were robustly reduced *(P < 0.05, P < 0.01, P < 0.001)* (Fig. 6f,g), which confirmed the suppression of pyroptosis. In summary, our results showed that ER stress elicited by PA contributed to the NLRP-inflammasome mediated activation of pyroptosis, and this process was blunted by ER stress inhibitor or OA.

### High-fat diet induced ER stress-mediated pyroptosis and olive oil ameliorated this effect in a rat NAFLD model

**Fig. 7:**
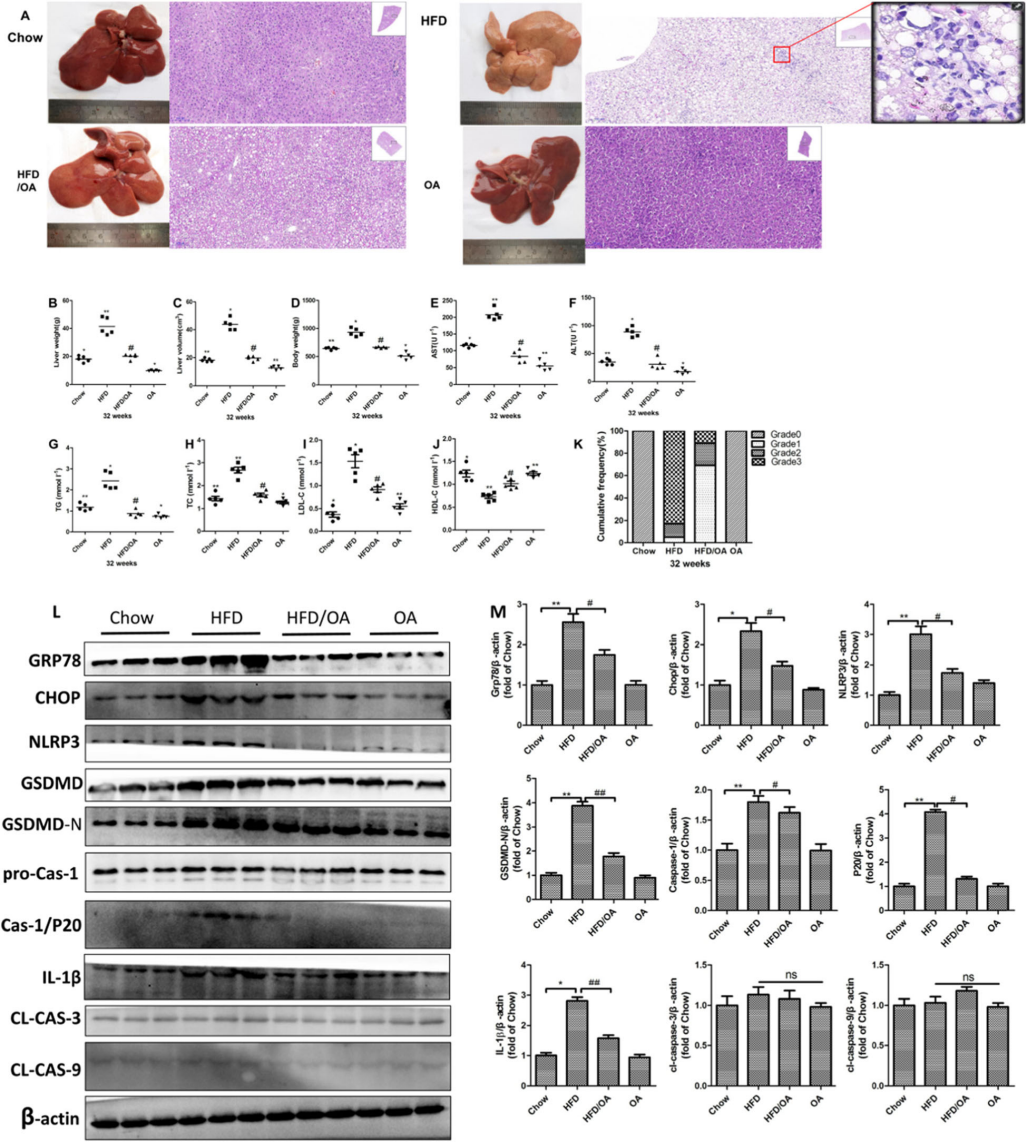
High-fat diet-induced ER stress-mediated pyroptosis and olive oil ameliorated this effect in a rat NAFLD model. **a**. H&E staining of liver sections from chow, HFD, HFD/OA and OA groups(*N* = 5). **b**. and **c**. Changes in liver weights and volume at the end of 32 w treatment. **d**. Changes in body weights of experimental animals from different groups. **e**-**j**. Serum levels of AST, AST, TG, TC,LDL-C and HDL-C. **k**. Scores of steatosis based on the representative H&E staining of liver sections from different groups. Grades of steatosis: 0 (no steatosis), 1 (< 33% steatosis), 2 (33–66% steatosis) or 3 (> 66% steatosis) (*n* = 5). **l**. and **m**. Representative western blots of ER stress and pyroptosis makers, and β-ACTIN was used as a protein-loading control. The data are presented as means ± SD for 3–5 biological replicates; *P < 0.05, **P < 0.01, and ***P < 0.001vs. Chow; #*P* < 0.05, ##*P* < 0.01, vs. HFD;ns no significant differences between two connected groups

Long term excessive uptake of saturated fatty acids is reported to induce NAFLD in either clinical cohorts or animal experiments. In the present study, we aimed to feed SD rats with HFD for 16 w to develop steatosis, and we half replaced the fat with olive oil in the diet for the next 16 w, producing a mix diet with both saturated and unsaturated fatty acids to mimic the in vitro experimental setting in which PA and OA interacted together. Our results showed that after 32 w HFD, the liver turned much paler and lacked lustre than others groups (Fig. 7a). The blood biochemistry indicated that the plasma levels of ALT, AST, TG, TC, LDL-C and HDL-C were significantly changed in HFD group, indicating the development of hyperlipidemia and abnormality of liver function. Interestingly, half replacement effectively ameliorated hyperlipidemia and decreased AST and ALT levels *(P < 0.05, P < 0.01)* (Fig. 7e-j). The histopathological examination of liver tissues showed that hepatocytes from HFD group exhibited greatly a zonal microvesicular steatosis, with much ballooning degeneration, and obvious inflammatory cells infiltration (Fig. 7a). To clearly and accurately describe the histopathological changes in all the experimental groups, we did the semiquantitative evaluation of the hepatic lesions based on the HE staining according to the scoring system and summarized the data in Fig. 7k. Chow diet and Olive oil groups did not present constitutive fatty livers. The majority of HFD rats presented grade-3 steatosis. However, after half replacement of HFD with olive oil, the animals mostly developed grade-1 microvesicular steatosis with much less liver weights and volumes, and particularly the inflammatory cells and necrotic cells were barely identified (Fig. 7b-d).

Furthermore, we investigated the effects of olive oil supplement on ER stress and pyroptosis of hepatocytes in NAFLD rats. We found that long term HFD significantly facilitated the protein expressions of both ER stress markers and pyroptosis markers including GRP78,CHOP(ER stress markers), NLRP3, GSDMD/−N, Caspase-1, p20, and IL-1 beta (pyroptosis markers), suggesting that ER stress and pyroptosis are important pathological process of NAFLD. Interestingly, only olive oil showed no impact on them. Notably, the HFD/Olive oil group displayed decreased GRP78 and CHOP levels upon ER stress, and the most distinct down-regulations presented on NLRP3, GSDMD/−N, Caspase-1, p20, and IL-1 beta for pyroptosis *(P < 0.05,P < 0.01)*(Fig. 7l-m), which confirmed the our findings of OA in HepG2 cells.

Overall, these results revealed that long term HFD feeding resulted in the development of NAFLD, and the activation of hepatic ER stress and pyroptosis in rats. Olive oil treatment (HFD/olive oil) effectively abolished these deleterious effects, implying its potential to alleviate HFD induced pyroptosis along with ER stress in NAFLD.

## Discussion

NAFLD represents a major health problem worldwide in the last decade. However, there are presently so limited pharmacologic treatments for it, particularly when NAFLD has progressed to NASH. Therefore, accumulating clinical evidence strongly supports that changes in diet and increase of physical activity can benefit NAFLD patients [23]. It is well demonstrated that the Mediterranean diet, rich of monounsaturated fatty acid, can reduce liver fat even without weight loss, so it is a greatly recommended dietary pattern for NAFLD. In consistence with other reports, our previous study has demonstrated the great protective effects of monounsaturated OA against saturated FFAs induced hepatic lipotoxicity in both hepatocytes and NASH rats [24]. In the present study, we aimed to further explore the underlying mechanisms, and we demonstrated that OA was able to effectively suppress PA induced ER stress and downstream pyroptosis activation, implying a novel mechanism for monounsaturated fatty acids mediated protection for lipotoxicity.

Previous studies have highlighted that the excessive accumulation of lipids in liver may lead to irreparable lipotoxicity, which is a primary contributor to the development and progression of steatohepatitis. Several major mechanisms are involved in FFAs caused cellular damage, in which ER stress and inflammation are two important cell stress signals. Some key molecules are particularly interacted to make cross talk between ER stress and inflammation, which is causally responsible for final cell death.

The role of ER stress in the pathology of lipotoxicity is extensively discussed in a variety of diseases, including neurodegenerative diseases, diabetes and metabolic disorders, atherosclerosis, cancer, as well as renal and lung diseases [25–28]. Many cellular perturbations can lead to the accumulation of unfolded or misfolded proteins inside the ER. When the UPR is insufficient to handle the unfolded protein load, cells undergo apoptosis. The reported downstream molecular mechanisms include activation of CHOP, which is induced via PERK and ATF4 and plays a master role in cell stress induced cell damage [29]. An interesting molecule in PA triggered ER stress is BIP (also named GRP78), a famous ER chaperone. It is well known that ER stress is initiated by the dissociation of BIP from the ER transducers PERK, IRE1, and ATF6, resulting in their activation [30], so GPR78 is recognized as an important molecular marker when the activation of ER stress. In agreement with many previous reports, including our previous results, we demonstrated that ER stress were remarkably elicited by saturated fatty acids in either HepG2 cells or HFD rats, evidenced by facilitated expression of CHOP and GRP78 (Figs. 4 and 7), and OA or olive oil was able to effectively suppress both PA and chemical ER stressor (TM) induced expressions of ER stress markers. Based on literatures, the widely studied downstream signals of ER stress are related to apoptosis, particularly lipoapoptosis. The associated mechanisms include that CHOP represses the antioxidant genes and induces growth arrest and DNA damage-inducible protein (GADD34) expression, which promotes dephosphorylation of eIF2α and subsequently increases oxidative stress to activate cell death. Alternatively, IRE1α can trigger apoptosis via activation of caspase molecules and pathways of TNF receptor-associated factor 2 (TRAF2) and c-Jun N-terminal kinase (JNK) [31]. However, in the present study, we have not found significant cell apoptosis occurred in PA challenged HepG2 cells as well as liver samples of HFD rats (Figs. 2 and 7), which could not explain the link between the simulated ER stress and substantial deterioration of cell viability or cell function we observed in both in vitro and in vivo models. Taken that, we hypothesize that there is alternative pathways cross talking with ER stress and governing cell destiny.

Pyroptosis is a novel programmed cell death, and is identified as caspase-1 dependent and characterized by plasma-membrane rupture and release of proinflammatory intracellular contents IL-1 beta and IL-18, which is distinct from other forms of cell death, especially apoptosis that is characterized by nuclear and cytoplasmic condensation and is elicited via activation of a caspase cascade [32]. Because their characteristics are actually similar, how to morphologically identify apoptosis and pyroptosis becomes an extraordinary difficult problem. It was previously reported that the structure called“pyroptotic vesicle”was discovered in macrophages by using an electron microscope, but that is hard to found in parenchymal cells [33]. In addition, more reports present cells undergoing pyroptosis by using AO/EB staining or Hoechst 33342/PI staining, which allow the visualization of cell membrane rupture without nuclear condensation. Similarly, by the fluorescent microscope, we found that a major characteristic of PA elicited pyroptotic cells is swelling and prominent nuclei, with simultaneously massive red fluorescence (EB) entering into cells. One the other hand, apoptotic cells were characterized by red fluorescence majorly surrounding the cell membrane, suggesting less membrane rupture occurred, which help us to distinguish pyroptosis from apoptosis (Fig. 3f). So, consistent to the gene or protein expression and FCM results, the morphological analysis also supported that PA exposure profoundly elicited pyroptosis rather than apoptosis, and OA was able to abrogate this effect.

In the present study, we demonstrated that ER stressor (TM) significantly increased both gene and protein expressions of pyroptotic markers NLRP3 and GSDMD, but no significant changes have been found on the expressions of caspase-1 and IL-1 beta in HepG2 cells (Fig. 5c, d). It is disagreeable to the previous report, showing increased levels of caspase-1 and IL-1beta in the livers as well as isolated primary hepatocytes of TM-treated mice [34]. Nevertheless, they also reported that IL-1 beta has tremendous raised in the livers of HFD-treated rats, which is consistent with our results (Fig. 7l). As we all know, for an integral pyroptosis, the release of mature IL-1 beta is processed by three major steps: the expression of pro-IL-1 beta by activating TLR2/4 pathway, then the precursors are cut by cleaved-caspase-1, and finally matured IL-1 beta was released through GSDMD mediated-pore. These results suggested that simple ER stress, provoked by ER stressors only, was able to induce NLRP3 inflammasome, but unable to promote pro-IL-1 beta and pro-caspase-1 accumulation in HepG2 cells, probably due to lacking the activation of TLR4 pathway [35], which may be mechanistically different with PA induced ER stress and pyroptosis. An alternative explanation might be that ER stress is able to regulate GSDMD directly, resulting in membrane perforation, but the potential mechanism is expected to be further explored.

## Conclusions

In summary, our study demonstrated that oleic acid, a monounsaturated fatty acid, was able to alleviate palmitic acid induced hepatic lipotoxicity both in vitro and in vivo. In HepG2 cells, for the first time, we demonstrated that OA addition robustly alleviated the PA caused pyroptosis, an ER stress triggered and NLRP3 inflammasome-mediated death form. It resulted in effective restoration of cell viability. In addition, we convinced the protective role of OA in the animal model of HFD rats. We found that exclusive olive oil supplementation had no detrimental effects on liver. Half replacement, in other word, the combined diet of saturated and monounsaturated FFAs, was able to effectively blunt the progression of NASH, and olive oil mediated inhibition of ER stress and downstream pyroptosis may play a critical role in the underlying mechanisms.

## Data Availability

All data generated or analyzed during this study are included in this published article or are available from the corresponding author on reasonable request.

## Abbreviations

ALT: Alanine transaminase
AO/EB: Acridine orange and ethidium bromide
ASC: Apoptosis-associated speck-like protein containing card
AST: Aspartate aminotransferase
BIP/GRP78: Binding-immunoglobulin protein/Glucose-regulated protein 78
BSA: Bovine serum albumin
CCK8: Cell Counting Kit-8
CHOP: Transcription factor CCAAT/−enhancer-binding protein homologous protein
ER: Endoplasmic reticulum
FBS: Fetal bovine serum
FCM: Flow cytometry
FFA: Free fatty acids
GSDMD: Gasdermin D
HDL-C: High-density lipoprotein cholesterol
HFD: High fat diet
IRE-1: Inositol- requiring enzyme 1
LDL-C: Low-density lipoprotein cholesterol
MUFA: Monounsaturated fatty acids
NAFLD: Non-alcoholic fatty liver disease
NASH: Non-alcoholic steatohepatitis
NLPR3: NOD-like receptor family pyrin domain containing 3
OA: Oleic acid
PA: Palmitic acid
SFA: Saturated fatty acids
TC: Total cholesterol
TG: Triglyceride
Thp: Thapsigargin
TM: Tunicamycin
TNF-α: Tumor necrosis factor alpha
TUDCA: Tauroursodeoxycholic acid
UPR: Unfolded protein response
Xbp-1: X-Box binding protein 1

## References

[1] Michelotti GA, Machado MV, Diehl AM (2013). NAFLD, NASH and liver cancer. Nat Rev Gastroenterol Hepatol.

[2] Mittal S, El-Serag HB, Sada YH (2016). Hepatocellular carcinoma in the absence of cirrhosis in United States veterans is associated with nonalcoholic fatty liver disease. Clin Gastroenterol Hepatol.

[3] Younossi ZM (2019). Non-alcoholic fatty liver disease–a global public health perspective. J Hepatol.

[4] Zhang J, et al. ER Stress-induced Inflammasome Activation Contributes to Hepatic Inflammation and Steatosis. J Clin Cellular Immunol. 2016;7(5):457–66. 10.4172/2155-9899.1000457 PMC5146989 27942420

[5] Eguchi A, Wree A, Feldstein AE (2014). Biomarkers of liver cell death. J Hepatol.

[6] Lerner AG, Upton J-P, Praveen PVK (2012). IRE1a Induces Thioredoxin-Interacting Protein to Activate the NLRP3 Inflammasome and Promote Programmed Cell Death under Irremediable ER Stress. Cell Metab.

[7] Fink SL, Cookson BT (2005). Apoptosis, Pyroptosis, and necrosis: mechanistic description of dead and dying eukaryotic cells. Infect Immun.

[8] Juarez-Hernandez E, Chavez-Tapia NC, Uribe M (2016). Role of bioactive fatty acids in nonalcoholic fatty liver disease. Nutr J.

[9] Barlow J, Hirschberg Jensen V, Jastroch M (2016). Palmitateinduced impairment of glucose-stimulated insulin secretion precedes mitochondrial dysfunction in mouse pancreatic islets. Biochem J.

[10] Lee JY, Cho HK, Kwon YH (2010). Palmitate induces insulin resistance without significant intracellular triglyceride accumulation in HepG2 cells. Metabolism.

[11] Schenkel LC, Bakovic M (2014). Palmitic acid and oleic acid differentially regulate choline transporter-like 1 levels and glycerolipid metabolism in skeletal muscle cells. Lipids.

[12] Ahn JH, Kim MH, Kwon HJ (2013). Protective effects of oleic acid against palmitic acid-induced apoptosis in pancreatic AR42J cells and its mechanisms. Korean J Physiol Pharmacol.

[13] Pareja A, Tinahones FJ, Soriguer FJ (1997). Unsaturated fatty acids alter the insulin secretion response of the islets of Langer hans in vitro. Diabetes Res Clin Pract.

[14] Sato K, Arai H, Miyazawa Y (2008). Palatinose and oleic acid act together to prevent pancreatic islet disruption in nondiabetic obese Zucker rats. J Med Investig.

[15] Morgan NG, Dhayal S (2010). Unsaturated fatty acids as cytoprotective agents in the pancreatic beta-cell. Prostaglandins Leukot Essent Fat Acids.

[16] Schwingshackl L, Strasser B, Hoffmann G (2011). Effects of monounsaturated fatty acids on glycaemic control in patients with abnormal glucose metabolism: a systematic review and metaanalysis. Ann Nutr Metab.

[17] de Barros CR, Cezaretto A, Curti ML (2014). Realistic changes in monounsaturated fatty acids and soluble fibers are able to improve glucose metabolism. Diabetol Metab Syndr.

[18] Schwingshackl L, Hoffmann G (2012). Monounsaturated fatty acids and risk of cardiovascular disease: synopsis of the evidence available from systematic reviews and meta-analyses. Nutrients.

[19] Joris PJ, Mensink RP (2016). Role of cis-monounsaturated fatty acids in the prevention of coronary heart disease. Curr Atheroscler Rep.

[20] Nigam P, Bhatt S, Misra A (2014). Effect of a 6-month intervention with cooking oils containing a high concentration of monounsaturated fatty acids (olive and canola oils) compared with control oil in male Asian Indians with nonalcoholic fatty liver disease. Diabetes Technol Ther.

[21] Assy N, Nassar F, Nasser G (2009). Olive oil consumption and non-alcoholic fatty liver disease. World J Gastroenterol.

[22] .Elizabeth M. Brunt, M.D. Nonalcoholic Steatohepatitis:Definition and Pathology.seminars in liver disease.21,1 2001. 10.1055/s-2001-12925 11296695

[23] de la Roche M (2018). Trafficking of cholesterol to the ER is required for NLRP3 inflammasome activation. J Cell Biol.

[24] Liu XH, Zeng X, Chen XM (2019). Oleic acid protects insulin-secreting INS-1E cells against palmitic acid-induced lipotoxicity along with an amelioration of ER stress. Endocrine.

[25] Xuanming C, Li LZ (2017). Oleic acid protects saturated fatty acid mediated lipotoxicity in hepatocytes and rat of non-alcoholic steatohepatitis.Lfs.

[26] Hotamisligil GS (2010). Endoplasmic reticulum stress and the inflammatory basis of metabolic disease. Cell.

[27] Cunard R (2017). Endoplasmic reticulum stress, a driver or an innocent bystander in endothelial dysfunction associated with hypertension?. Curr Hypertens Rep.

[28] Fu S, Watkins SM, Hotamisligil GS (2012). The role of endoplasmic reticulum in hepatic lipid homeostasis and stress signaling. Cell Metab.

[29] Hetz C, Chevet E, Harding HP (2013). Targeting the unfolded protein response in disease. Nat Rev Drug Discov.

[30] Lindholm D, Korhonen L, Eriksson O (2017). Recent insights into the role of unfolded protein response in ER stress in health and disease. Front Cell Dev Biol.

[31] Clark AL, Urano F (2016). Endoplasmic reticulum stress in beta cells and autoimmune diabetes. Curr Opin Immunol.

[32] Cybulsky AV (2017). Endoplasmic reticulum stress, the unfolded protein response and autophagy in kidney diseases. Nat RevNephrol.

[33] He W (2015). Gasdermin D is an executor of pyroptosis and required for interleukin-1β secretion. Cell Res.

[34] Schroder K, Zhou R, Tschopp J (2010). The NLRP3 inflammasome: a sensor for metabolic danger?. Science.

[35] Paloque L, Perez-Berezo T, Abot A, et al. Polyunsaturated fatty acid metabolites biosynthesis in Leishmania and role in parasite_host interaction. J Lipid Res. 2019;60(3):636–47. 10.1194/jlr.M091736 PMC6399491 30626624

