# Correction to: Oleic acid ameliorates palmitic acid induced hepatocellular lipotoxicity by inhibition of ER stress and pyroptosis

**DOI:** 10.1186/s12986-020-00438-y

**Published:** 2020-03-12

**Authors:** Xin Zeng, Min Zhu, Xiaohong Liu, Xuanmin Chen, Yujia Yuan, Lan Li, Jingping Liu, Yanrong Lu, Jingqiu Cheng, Younan Chen

**Affiliations:** Key Laboratory of Transplant Engineering and Immunology, NHFPC, Regenerative Medicine Research Center, West China Hospital, Sichuan University, No. 1, Keyuan 4th Road, Gao Peng Street, Chengdu, Sichuan 610041 People’s Republic of China

**Correction to: Nutr Metab (2020) 17:11**

**https://doi.org/10.1186/s12986-020-0434-8**

The original article [1] possessed a minor typo in first author, Xin Zeng’s name which has since been corrected.
